# TAP Mediates Import of *Mycobacterium tuberculosis*-Derived Peptides into Phagosomes and Facilitates Loading onto HLA-I

**DOI:** 10.1371/journal.pone.0079571

**Published:** 2013-11-11

**Authors:** Melanie J. Harriff, Sven Burgdorf, Christian Kurts, Emmanuel J. H. J. Wiertz, Deborah A. Lewinsohn, David M. Lewinsohn

**Affiliations:** 1 Portland VA Medical Center, Portland, Oregon, United States of America; 2 Department of Pulmonary and Critical Care Medicine, Oregon Health & Sciences University, Portland, Oregon, United States of America; 3 Life and Medical Sciences (LIMES) Institute, University of Bonn, Bonn, Germany; 4 Institutes of Molecular Medicine and Experimental Immunology (IMMEI), University of Bonn, Bonn, Germany; 5 University Medical Center Utrecht, Utrecht, The Netherlands; 6 Leiden University Medical Center, Leiden, The Netherlands; 7 Department of Pediatrics, Oregon Health & Sciences University, Portland, Oregon, United States of America; 8 Department of Molecular Microbiology and Immunology, Oregon Health & Sciences University, Portland, Oregon, United States of America; University of Maryland, United States of America

## Abstract

Processing and presentation of antigen on MHC-I class I molecules serves to present peptides derived from cytosolic proteins to CD8^+^ T cells. Infection with bacteria that remain in phagosomal compartments, such as *Mycobacterium tuberculosis* (Mtb), provides a challenge to this immune recognition as bacterial proteins are segregated from the cytosol. Previously we identified the Mtb phagosome itself as an organelle capable of loading MHC Class I molecules with Mtb antigens. Here, we find that the TAP transporter, responsible for importing peptides into the ER for loading in Class I molecules, is both present and functional in Mtb phagosomes. Furthermore, we describe a novel peptide reagent, representing the N-terminal domain of the bovine herpes virus UL49.5 protein, which is capable of specifically inhibiting the lumenal face of TAP. Together, these results provide insight into the mechanism by which peptides from intra-phagosomal pathogens are loaded onto Class I molecules.

## Introduction

Tuberculosis (TB) remains a global health concern, whose impact is compounded by the emergence of multiple drug-resistant strains and incidence of co-infection with HIV. Following aerosol exposure, *Mycobacterium tuberculosis* (Mtb) can be taken up in the lung by resident macrophages and dendritic cells (DC) where it resides in a phagosomal compartment. The ability to control intracellular growth of Mtb is dependent upon acquisition of a robust Th1-type adaptive immune response. CD4^+^ T cells play an important role in this process, but CD8^+^ T cells are also essential to contain Mtb because of their unique ability to recognize intracellular infection [1]. Although it is known that the phagosome is a component of the MHC-II antigen processing pathway [2], the mechanisms by which Mtb antigens are processed and presented on MHC-I molecules are less well understood. In contrast to a viral infection, where viral proteins are abundant in the cytosol and readily available to Class I antigen processing machinery, mycobacterial antigens found within the phagosome pose unique challenges for immune recognition. Consequently, multiple mechanisms have been described for the recognition of these antigens within the phagosome [3].

Studies using latex or iron bead containing phagosomes have defined several pathways by which particulate antigens can be processed and presented on MHC-I molecules [4]. For some antigens, processing can be characterized as “cytosolic” as processing requires access of the protein to the cytosol, proteasomal degradation, transport of peptide fragments into the endoplasmic reticulum (ER) by the transporter associated with antigen processing (TAP), and loading of peptides onto Class I molecules in the ER [5]. Other antigens are processed by a vacuolar pathway, in which they are degraded by vacuolar proteases such as Cathepsin S, and never access the cytosol prior to being displayed on the cell surface [6], [7]. Finally, a third mechanism has identified the phagosome as a compartment capable of cross presenting exogenous antigens [8]–[10]. Although understanding of Mtb antigen processing and presentation on MHC-I molecules is incomplete, studies have revealed that Mtb antigens can be processed and presented by both cytosolic and vacuolar pathways [11]–[14], as well as by the Mtb phagosome [15].

In this regard, we previously identified the Mtb phagosome as an organelle that contains molecules involved in antigen processing and presentation, and demonstrated that loaded HLA-E molecules are present in these phagosomes [15]. Additionally we found that a major pathway of MHC-I Mtb antigen processing for secreted proteins is TAP-dependent [15]–[17]. However, a direct role for TAP in phagosomal loading of Mtb antigens has not yet been demonstrated. Here, using a novel peptide reagent representing the lumenal domain of the BHV-I encoded TAP inhibitor, UL49.5, we investigate the role of phagosomal TAP with regard to the import of peptides. We demonstrate that Mtb peptides are imported into the Mtb phagosome in a TAP-dependent manner and that phagosomal TAP is required for loading of Mtb antigens onto an Mtb-specific HLA-E T cell clone. These data further implicate the Mtb phagosome in the presentation of HLA-E restricted Mtb antigens, and are the first demonstration that phagosomal TAP plays a role in loading of these antigens.

## Results

### TAP is present in highly pure Mtb phagosomes

**Figure 1:**
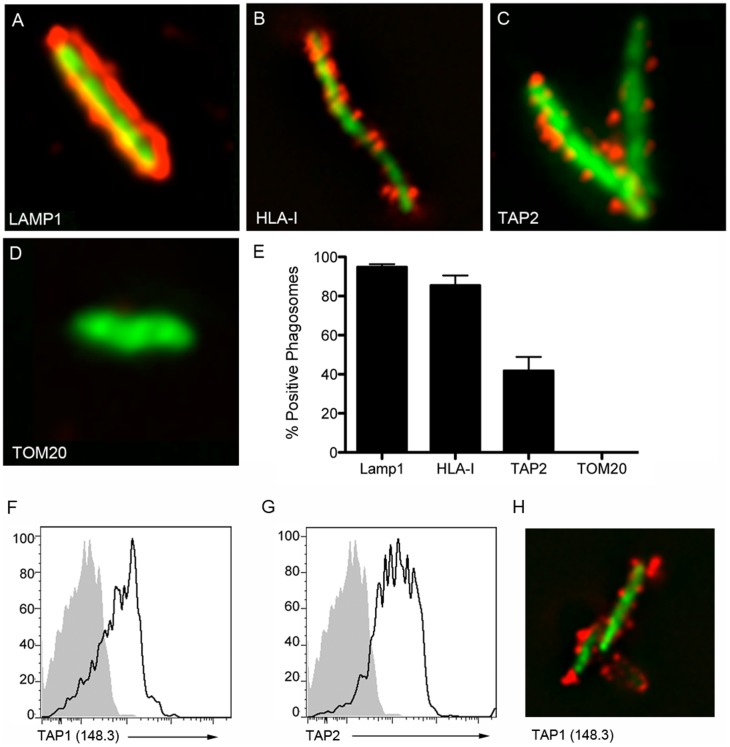
TAP is present in highly pure Mtb phagosomes. Mtb-containing phagosomes were stained with antibodies against A) LAMP1; B) HLA-I; C) TAP2 (2.17); or D) TOM20, a Golgi marker used as a negative control; and imaged on a high-resolution wide field Core DV system. One 0.2 um Z-section is shown. E) Mean and SEM of percentage of positive phagosomes (>100 observations) for each marker is shown. F-H) Mtb-containing phagosomes were stained with non-commercial antibodies against TAP1 or TAP2, and analyzed by flow organellometry (F-G) or fluorescence microscopy (H).

Previously, we described a method to isolate highly pure Mtb-containing phagosomes from human DC [15]. Flow organellometry of these phagosomes demonstrated the presence of both TAP2 and HLA-I. To visually determine the proportion of Mtb phagosomes containing these markers, fluorescence microscopy was performed. Consistent with previous observations [15], [18], [19], Lamp1 completely surrounded the Mtb (Figure 1A), while HLA-I was observed in discrete areas throughout the phagosomal membrane (Figure 1B). Using the same antibody against TAP2 used by Grotzke *et al.* (2009), TAP2 was also observed in discrete areas throughout the phagosomal membrane (Figure 1C). The Golgi marker, TOM20, was used as a negative control and was not present on Mtb phagosomes (Figure 1D). At least 100 Mtb phagosomes were assessed for the presence of Lamp11, HLA-I, TAP2, and TOM20, and the percentage of positive phagosomes was calculated. Lamp1 and HLA-I were observed in over 90% of the phagosomes, while TAP2 was observed in 45% of the phagosomes.

Because the percentage of TAP2 positive phagosomes was greater than previously observed by flow organellometry, we further analyzed Mtb-containing phagosomes for the presence of TAP using two additional antibodies, α-TAP1 (148.3) and α-TAP2 (435.3). Flow organellometry using these antibodies indicates that most of the phagosomes are TAP positive (Figure 1F-G). These results were confirmed by assessing phagosomes by fluorescence microscopy as described above. The pattern of distribution of TAP in discrete areas of the phagosome when visualized with these antibodies is similar to what was seen with the TAP2.17 antibody (Figure 1H). These results suggest that we have previously underestimated the number of Mtb phagosomes containing TAP.

### The UL49.5 peptide inhibits TAP-dependent antigen processing and presentation

**Figure 2:**
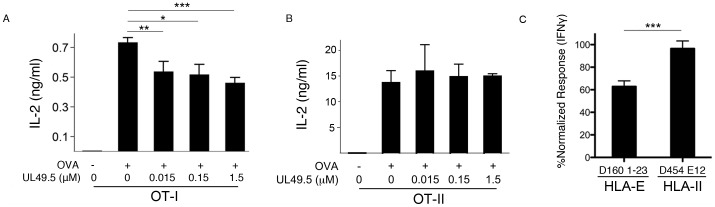
UL49.5 peptide inhibits TAP-dependent presentation of antigen to CD8^+^ T cell clones. A-B) Murine DCs were incubated with 0.5 mg/ml OVA and the indicated concentrations of the UL49.5-NP. DCs were then co-cultured with OT-I T (A) or OT-II (B) cells and supernatants were analyzed for IL-2 by ELISA. Shown is the mean and standard error of 2 independent experiments. * p = 0.07; ** p = 0.01; *** p = 0.007 (Student's two-tailed t test). C) Human monocyte derived DC were incubated for 1 hr with UL49.5-NP (5 uM) or UL49.5-SCR (5 uM) peptides and then infected with H37Rv-eGFP (MOI = 10) overnight. IFNγ production by the TAP-dependent HLA-E-restricted T cell clone D160 1-23 or the TAP-independent Class II clone D454 E12 was assessed by ELISPOT. IFNγ response by each T cell clone following UL49.5-NP treatment was normalized to the response in the presence of UL49.5-SCR. Shown is the mean response and standard error from at least 4 independent experiments. *** denotes significantly reduced IFNγ production by D160 1–23 in the presence of UL49.5-NP compared to D454 E12 (Student's two-tailed t test, p<0.001).

Viruses have evolved numerous mechanisms aimed at inhibiting antigen presentation, many of which target TAP specifically [20]–[22]. We previously used one of these inhibitors, ICP47, to show that presentation of Mtb antigen on HLA-E is TAP-dependent [15]. Most of these inhibitors block TAP in both the ER as well as the phagosome, limiting our ability to discern a direct role for TAP in the Mtb phagosome. To investigate the role of TAP in Mtb-containing phagosomes, we utilized the multifunctional properties of a different TAP inhibitor, the BHV-1 UL49.5 protein. The N-terminal domain of UL49.5 blocks peptide uptake by binding the lumenal face of TAP and inducing a conformational arrest of the TAP complex, while the C-terminal domain of UL49.5 induces degradation of TAP [23]. We sought to replicate the lumen-specific inhibition through the design of a synthetic peptide representing the N-terminal domain (UL49.5-NP). We first evaluated the general ability of the synthetic UL49.5-NP to inhibit TAP function. Using OVA protein, we assessed TAP-dependent and TAP-independent antigen-specific cytokine production by CD8^+^ and CD4^+^ T cells, respectively. Incubation of OVA-pulsed murine DC with UL49.5-NP significantly reduced the TAP-dependent, OVA-specific IL-2 production by primary naive OT-1 T cells in a concentration-dependent fashion (Figure 2A), with no effect on TAP-independent OT-II T cells (Figure 2B).

We then determined the ability of exogenously delivered synthetic UL49.5-NP to inhibit TAP-dependent loading of antigens in the context of Mtb infection by assessing the response of two well-characterized Mtb-specific T cell clones. The HLA-E-restricted D160 1–23 clone is stimulated through a TAP-dependent mechanism [15], while the D454 E12 Class II T cell clone is stimulated through a TAP-independent mechanism. IFN-γ production by the D160 1–23 T cell clone in response to human DC infected with Mtb was analyzed in the presence of UL49.5-NP or a scrambled control peptide (UL49.5-SCR). As shown in Figure 2C, addition of the UL49.5-NP to DC prior to Mtb infection inhibited recognition of these cells by D160 1–23 by 38% compared to addition of UL49.5-SCR. No effect was seen on the recognition of these cells by the D454 E12 CD4^+^ T cell clone, arguing against simple peptide toxicity. These results indicate that the exogenously delivered synthetic UL49.5-NP retains the ability to inhibit TAP-mediated peptide transport.

### Phagosomal TAP transports peptides into the lumen

**Figure 3:**
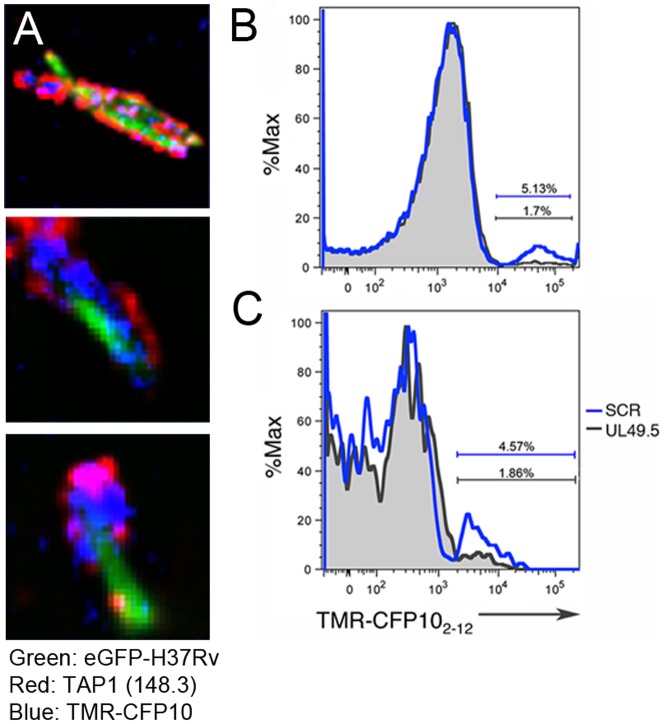
Phagosomal TAP transports peptides into the lumen. A) Isolated GFP-Mtb-phagosomes were incubated with TMR-conjugated CFP10_2–12_ peptide (blue), then fixed and stained with anti-TAP1 (red) and analyzed by fluorescence microscopy. B) TMR-conjugated CFP10_2–12_ peptide (500 nM) and UL49.5-NP (10 uM) or UL49.5-SCR (10 uM) were added simultaneously to isolated Mtb phagosomes and incubated at 37° C for 30 min. Washed and fixed phagosomes were analyzed by FACS. FACS plot is representative of three independent experiments. C) Prior to infection, Mtb was coupled to UL49.5-NP or UL49.5-SCR to target the inhibitor specifically to the Mtb phagosome. Isolated phagosomes were then incubated with TMR-conjugated CFP10_2–12_ peptide as indicated above. FACS plot is representative of two independent experiments.

ER-associated TAP transports proteasomally-digested peptides from the cytosol into the lumen. Whether or not TAP plays a similar role in the Mtb phagosome is not known. Therefore, we used UL49.5-NP to determine if the TAP molecules observed in the Mtb phagosome membrane are functional. Highly pure Mtb phagosomes were incubated with a fluorescently labeled peptide derived from the Mtb CFP10 protein (TMR-CFP10_2–12_). As shown in Figure 3A, TMR-CFP10_2–12_ peptide can be visualized by microscopy in Mtb-containing phagosomes. Fluorescence microscopy indicated that nearly all phagosomes contained fluorescent CFP10_2–12_ peptide; however, we also note considerable heterogeneity in peptide uptake. To delineate a role for TAP, the peptide uptake assay was performed in the presence of UL49.5-NP or UL49.5-SCR. Using flow cytometric analysis, we identified an average of 6.3 (+/−1.9)% of Mtb-phagosomes incubated with UL49.5-SCR that exhibited robust CFP10_2-12_ peptide uptake, while addition of UL49.5-NP resulted in 58.2 (+/−12)% reduction on average of peptide uptake (Figure 3B).

To provide further evidence of the specific functionality of phagosomal TAP, UL49.5-NP or UL49.5-SCR were synthesized with an amine-reactive biotin moiety on the N-terminus, and then coupled by biotin-streptavidin linkage to Mtb prior to infection. Highly pure Mtb-containing phagosomes were isolated and incubated with TMR-CFP10_2–12_ as described above. The direct coupling of UL49.5-NP to Mtb prior to infection also resulted in a decrease in uptake of peptide from 4.5 (+/−0.1)% to 1.7 (+/−0.2)% compared to UL49.5-SCR (Figure 3C), further supporting a functional role for TAP in the Mtb phagosome.

### UL49.5 peptide blocks presentation of Mtb antigen loaded in the phagosome

**Figure 4:**
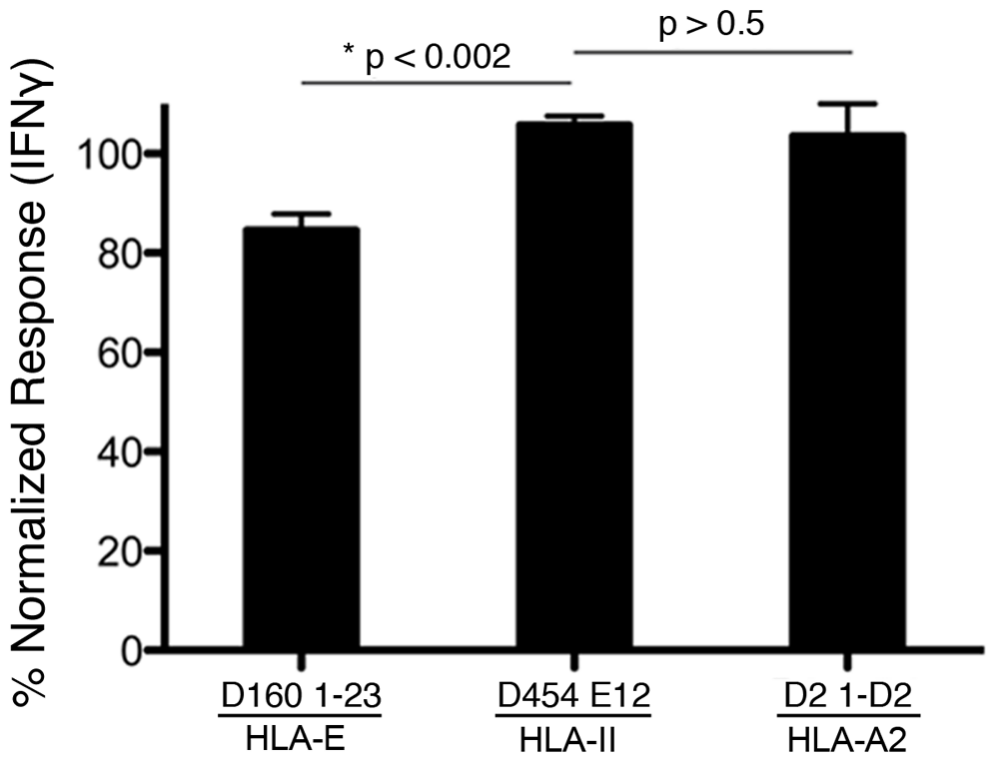
UL49.5 peptide blocks presentation of Mtb antigen loaded in the phagosome. H37Rv Mtb was linked to UL49.5-NP or UL49.5-SCR through biotin-steptavidin interactions. DC were then infected overnight with UL49.5-NP-linked or UL49.5-SCR-linked Mtb. For the D2 1-D2 clone, DC were also co-infected with a vaccinia virus expressing the D2 1-D2 epitope from HCMV pp65. IFNγ production by D160 1–23 (HLA-E), D454 E12 (Class II), or D2 1-D2 (HLA-A2) was assessed by ELISPOT assay. IFNγ response for each T cell clone was normalized to the scrambled peptide control. Shown is the mean response and standard error from 7 independent experiments (D160 1–23) or 2 independent experiments (D454E12, D2 1-D2). The asterisk denotes significantly reduced IFNγ production by D160 1–23 in the presence of UL49.5 compared to D454 E12 (Student's two-tailed t test, p<0.002).

Having demonstrated that UL49.5-NP can inhibit the uptake of peptide into isolated Mtb phagosomes, we then asked whether we could directly demonstrate a role for phagosomal TAP in the processing and presentation of Mtb-derived antigens. Specifically, we coupled UL49.5-NP to the Mtb by direct biotin-streptavidin linkage of UL49.5-NP or UL49.5-SCR to Mtb prior to infection. DC infected with UL49.5-NP- or UL49.5-SCR-coupled Mtb were then used as antigen presenting cells in an IFN-γ Elispot assay with TAP-dependent and TAP-independent T cell clones. Coupling of UL49.5-NP directly to the Mtb resulted in a modest (16%) but highly significant decrease in IFN-γ release by D160 1–23, in comparison to UL49.5-SCR coupled Mtb, while recognition by D454 E12 was not affected (Figure 4). To show that the effect of the UL49.5 peptide was localized to the phagosome, and not altering TAP activity in the ER, we also measured presentation of virally expressed endogenous antigen. DC were co-infected with vaccinia virus expressing HCMV pp65 and UL49.5-NP- or UL49.5-SCR-coupled Mtb. The response by the pp65-specific CD8^+^ T cell clone, D2 1-D2, was not changed in the presence of UL49.5-NP-coupled Mtb (Figure 4). These data indicate that the UL49.5-NP does not access the cytsosol and ER-associated TAP, and confirm the specificity of the UL49.5 peptide for phagosomal TAP. This result demonstrates that phagosomal TAP plays a role in processing and presentation of Mtb peptide on Class I molecules.

## Discussion

By virtue of their dependence on intracellular replication, viruses have evolved a plethora of mechanisms to subvert immune recognition, one of which is reduced recognition by CD8^+^ T cells [21]. The fact that TAP is so frequently targeted suggests that this molecule is of central importance, and has provided critical tools to delineate the mechanisms of antigen processing and presentation [20], [22]. In this report, we find that, when administered as a synthetic peptide, the lumenal specific N-terminal domain of UL49.5 is sufficient to inhibit TAP function and subsequent activation of CD8^+^ T cells. This synthetic peptide reagent is therefore a useful tool to analyze the role of specific cellular compartments such as the phagosome in TAP-dependent antigen processing and presentation pathways.

Although viral proteins are both abundant and readily available for entry into the cytosolic antigen processing and presentation pathway, it is less clear how phagosomal proteins or particles gain access to this pathway. Redundant mechanisms have been described for the sampling of this environment for both classical and non-classical MHC molecules [3]. We have previously defined the Mtb phagosome as a competent antigen processing organelle in that TAP and HLA-I were observed, and the Mtb phagosome was sufficient for presentation to CD8^+^ T cells [15]. Additionally, we demonstrated that ICP47-mediated TAP inhibition diminished the presentation of Mtb-derived antigen. Because ICP47 inhibits the cytosolic face of TAP, we could not distinguish a role for TAP in the transport of peptides into the ER versus the phagosome. Here, we extended these results using a synthetically–derived peptide domain of a viral TAP inhibitor, UL49.5 (UL49.5-NP). We first demonstrate that this synthetic peptide is capable of inhibiting TAP-dependent antigen presentation. Furthermore, we establish that Mtb phagosomes contain TAP molecules and take up Class I peptides, and this uptake is blocked in the presence of UL49.5-NP. Finally, we directly confirm that phagosomal TAP is functional by coupling UL49.5-NP to Mtb prior to infection and demonstrating reduced TAP-dependent T cell responses. Thus, this study provides further confirmation and mechanistic resolution of the phagosome as a competent antigen processing organelle.

While we effectively inhibited peptide uptake into isolated Mtb phagosomes with UL49.5-NP, coupling of UL49.5-NP to Mtb resulted in a more modest decrease in TAP-dependent T cell recognition. Here, we consider several possibilities. First, there may be differences in the abundance and/or functionality of free UL49.5-NP compared to Mtb-conjugated UL49.5-NP. For example, the biotin-streptavidin coupling of UL49.5-NP to the Mtb could result in steric hindrance of UL49.5 function. Second, if UL49.5-NP binds to TAP molecules irreversibly, then new TAP molecules arriving to the phagosome may be left unblocked in the absence of additional UL49.5-NP. Alternately, the difference in degree of inhibition observed in these experiments could relate to the specific targeting of UL49.5 to the Mtb phagosome. We do not have an estimate of the proportion of HLA-E loading that occurs in the phagosome vs. the ER. In this regard, redundant mechanisms for loading Mtb antigens onto HLA-E would be expected to decrease the effect of specific targeting inhibition of phagosomal TAP.

One unexpected finding was the heterogeneity of the Mtb phagosomes with regard to the functionality of TAP. Prior work on the characterization of the Mtb phagosome has relied on biochemical techniques, such as mass spectrometry and Western blotting [24], and hence could not provide a direct analysis of individual phagosomes. To validate our previous findings [15], we obtained non-commercially available antibodies against TAP1 and TAP2 to assess the localization of TAP to the Mtb phagosome. Using these antibodies we were able to demonstrate the presence of TAP1 and TAP2 in nearly all phagosomes, suggesting the commercially available TAP2.17 antibody has a lower avidity. In the case of all antibodies tested, our work suggests, however, that while some molecules such as LAMP1 may be ubiquitous components of the phagosome, other molecules such as TAP and HLA-I are present in distinct areas of the phagosome. Furthermore, only a small proportion of the total phagosomes isolated from infected cells were able to import high import high levels of CFP10_2-12_ peptide to the lumen that we believe are necessary for flow cytometric detection. Interestingly, when assessed by fluorescence microscopy, all phagosomes had at least low levels of peptide. The molecular mechanisms underlying these differences are not known, but it is clear that the phagosome is a dynamic organelle [25], [26]. Hence, heterogeneity in the distribution and functionality of TAP molecules could reflect continual development and remodeling of the Mtb phagosome.

In conclusion, we have described a novel TAP-blocking reagent, UL49.5-NP, and have also demonstrated for the first time both the presence and functionality of TAP in the human Mtb phagosome. Processing and loading of antigen within the phagosome may facilitate the immune recognition of intracellular Mtb infection by CD8+ T cells, and hence contribute to the host response to persistent infection.

## Experimental Procedures

### Antibodies and reagents

All reagents were obtained from Sigma unless otherwise noted. Anti-TAP2 (TAP2.17, BD), Anti-TAP1 (148.3, a kind gift from P. Cresswell), anti-TAP2 [27] anti-TOM20 (29/Tom20, BD), anti-LAMP1 (H5G11, SCBT), anti-HLA-ABC (W6/32, Serotec), and goat anti-mouse IgG1-Alexa-568, GAM IgG2A-Alexa-568, or goat anti-mouse IgG-Alexa568 (Invitrogen) were used for fluorescence microscopy and flow cytometry. Peptides were synthesized and obtained from Genemed Synthesis or Dr. J.W. Drijfhout and W.E. Benckhuijsen, at the Department of Immunohematology and Blood Transfusion of Leiden University Medical Center: TMR-CFP10_2–12_ (N)-AEMK-(TMR)-TDAATLA-(C); Biotin-UL49.5 (N)-RDPLLDAMRREGAMDFWSAGCYARGVPLSEKKGSGGSGGS-B-(C); Biotin-SCR (N)-FWGAARSERDLKDSMRPLMAVPLDYGEGRCAKGSGGSGGS-B-(C).

### Bacteria, virus, and cells

Mtb H37Rv-eGFP (a kind gift from Joel Ernst) was grown in Middlebrook 7H9 broth (BD) supplemented with Middlebrook ADC (BD), 0.05% Tween-80, 0.5% glycerol, and kanamycin (50 ug/ml). Vaccinia virus expressing HCMV pp65 was provided by Stan Riddell. Human monocyte derived dendritic cells (DC) were generated from PBMC as described previously [15]. Bone marrow-derived murine DC were generated as described previously [28]. T cell clones were derived and expanded as previously described [15], [29]. D160 1–23 is an HLA-E restricted CD8+ T cell clone that responds to a component of the Mtb cell wall and D454 E12 is a CD4+ clone, specific to the Mtb CFP10 protein [15]. D2 1-D2 is an HLA-A2 restricted CD8^+^ T cell clone specific to an epitope from HCMV pp65 [29]. This study was conducted according to the principles expressed in the Declaration of Helsinki. The study was approved by the Institutional Review Board of Oregon Health and Sciences University (IRB00000186). All patients provided written informed consent for the collection of samples and subsequent analysis.

### Mtb coupling to UL49.5 and SCR peptides

H37Rv-eGFP was labeled with magnetic microbeads as described previously [15]. Where indicated, streptavidin bead-labeled bacteria were then incubated with biotin-linked UL49.5 or SCR peptides (25 uM) for 30 minutes at room temperature. Subsequent magnetic isolation of highly pure Mtb phagosomes from DC has been described previously [15].

### Fluorescence microscopy

Isolated Mtb phagosomes were fixed and stained with α-Lamp1 (1∶250), α-TAP2 (TAP2.17 1∶100), α-TAP1 (148.3 1∶1500), α-TAP2 (Wiertz 1∶50), α-TOM20 (1∶100), or α-HLA-ABC (1∶250), followed by GAM-IgG1-Alexa568 (1∶2000), GAM-IgG2A-Alexa568 (1∶2000), or GAM-IgG-Alexa568. Images were acquired on a high-resolution wide field Core DV system (Applied Precision ™) with a Nikon Coolsnap ES2 HQ. Each image was acquired as Z-stacks in a 256×256 format with a 100×1.42 NA Plan Apo N objective. Images were deconvolved with an optical transfer function using an iterative algorithm of 10 iterations.

### Peptide translocation assay

Isolated Mtb phagosomes were resuspended in cold ICT buffer (50 mM Hepes pH 7.0, 78 mM KCl, 4 mM MgCl2, 8.37 mM CaCl2, 10 mM EGTA, 1 mM DTT, 4 mg/ml BSA). With phagosomes on ice, the TMR-conjugated peptide (500 nM) was added to the samples. For data shown in Figure 3b, UL49.5 or SCR peptide (10 uM) was added simultaneously. For data shown in Figure 3c, phagosomes containing UL49.5- or SCR-coupled Mtb, generated as described above, were used in the assay. After incubation at 37 C for 30 min, samples were washed extensively on ice, then resuspended in 1% PFA for 15 min. Fixed phagosomes were stained with α-TAP1 (148.3 1∶1500) or α-TAP2 (Wiertz) and analyzed by FACS or fluorescence microscopy as described above.

### UL49.5-mediated inhibition of OVA-specific IL-2 production

Bone marrow-derived murine DC (2.5×10^5^) were incubated with 0.5 mg/ml OVA and the indicated concentrations of UL49.5 peptide. After 2 hours, DC were fixed with 0.008% glutaraldehyde for 3 min, washed extensively and co-cultured with OVA-specific OT-I or OT-II T cells (7×10^5^ cells/well). T cell activation was depicted by secretion of IL-2 in the supernatant by ELISA after another 18 h.

### UL49.5-mediated inhibition of Mtb-specific IFN-γ production

Human DC (2.5×10^6^) were infected overnight with H37Rv-eGFP. For experiments shown in Figure 2, biotin-UL49.5 (5 uM) or biotin-SCR (5 uM) peptides were added to DC 1 hour prior to infection. For experiments shown in Figure 4, DC were infected with UL49.5- or SCR-coupled Mtb, generated as described above. Where indicated, 2.3×10^7^ pfu VV expressing HCMV pp65 were added to the DC and incubated overnight after infection with UL49.5-labeled Mtb. DC were added to an IFN-γ ELISPOT plate and T cell clones were added in excess. After overnight incubation, IFNγ ELISPOT was performed as previously described [30].

### Statistical Analysis

Statistical significance of inhibition was determined using Student's two-tailed t test compared to control treated cells, unless otherwise indicated.
